# A Novel Approach for Monitoring the Volatile Metabolome in Biological Samples from Ruminants through Miniaturized Liquid–Liquid Extraction and Multiclass Gas Chromatography Analysis

**DOI:** 10.1021/acs.jafc.1c06662

**Published:** 2022-03-17

**Authors:** Liliana Cordeiro, Ana R. J. Cabrita, Hugo M. Oliveira, Margarida R. G. Maia, José A. Rodrigues, António J. M. Fonseca, Inês M. Valente

**Affiliations:** † REQUIMTE, LAQV, ICBAS, Instituto de Ciências Biomédicas Abel Salazar, Universidade do Porto, Rua Jorge Viterbo Ferreira, 228, 4050-313 Porto, Portugal; ‡ INL, International Iberian Nanotechnology Laboratory, Avenida Mestre José Veiga s/n, 4715-330 Braga, Portugal; § REQUIMTE, LAQV, Departamento de Química e Bioquímica, Faculdade de Ciências, Universidade do Porto, Rua do Campo Alegre 687, 4169-007 Porto, Portugal

**Keywords:** ruminant animals, salt-assisted liquid−liquid extraction, volatile compounds

## Abstract

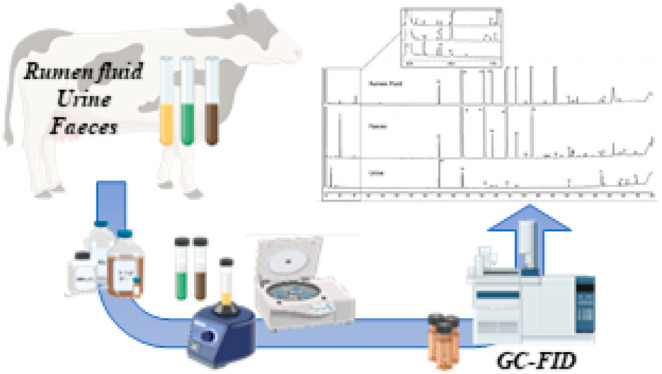

A straightforward and versatile methodology for the extraction of volatile metabolites in biological samples from ruminants for gas chromatography analysis is proposed. The methodology was applied in the determination of multiclass metabolites (short-chain fatty acids, aldehydes, alcohols, ketones, esters, phenols, and sulfides) in different analytical matrices (rumen fluid, urine, and feces) collected from Holstein cows. The 24 multiclass volatile metabolites reported in the different biological samples and their respective concentrations were critically discussed in the context of digestive physiology. Most detected compounds are derived from the rumen and lower gut fermentation of carbohydrates, proteins, and lipids or their metabolism, being consistent with the prior state of the art. The proposed method also takes advantage of the already existing tools in animal nutrition laboratories, providing a novel methodological ground that can generate relevant bioanalytical information with a significant impact on ruminant’s nutritional studies.

## Introduction

In ruminants, the digestion of feeds mainly occurs by anaerobic fermentation in the rumen, a forestomach that harbors a microbial population comprising bacteria, protozoa, fungi, archaea, and bacteriophages.^1^ The symbiosis between the ruminant animal and the rumen microbial population enables these animals to obtain nutrients from human nonedible feedstuffs and to transform them into high nutritional value products, such as meat and milk. The importance of the rumen microbiome and secondary metabolites formed during fermentation to the animal host is clearly illustrated by the fact that 70% of the metabolic energy utilized by the animal is obtained by the main fermentation metabolites, short-chain fatty acids (SCFA).^2^ Alongside with SCFA, a myriad of metabolites is formed in the rumen, including amino acids, peptides, proteins, sugars, fatty acids, lipids, other organic acids, and volatile compounds formed at much lower concentrations.^3−5^

Due to its huge impact on animal health and performance, monitoring the rumen function is crucial. This is normally achieved by analyzing the fermentation end-products, mainly SCFA and ammonia-N, of the rumen fluid. However, rumen sampling involves the use of invasive techniques such as oral intubation, ruminocentesis (i.e., puncture of the abdominal wall with a needle), and fitting a rumen cannula. The former two are unpleasant procedures for the animal, with rumen fluid often heavily contaminated with saliva in oral intubation,^6^ and limited volume of sample collected by ruminocentesis.^7^ Rumen sampling through ruminal cannula provide reliable samples, but requires skilled surgery and precludes its use in commercial farms.^7^ Blood analysis can also provide valuable information on the metabolism of the animal by monitoring of the levels of bicarbonate, β-hydroxybutyrate, cholesterol, and fatty acids, for example.^8,9^ Therefore, there is a need to characterize the metabolic profile of noninvasive biological samples, which can provide relevant information about the rumen function and the animal metabolism.

In this context, analytical methods that provide significant information about the metabolomic status of the animal in a simple and versatile fashion are critical tools. The current state of the art in the analysis of biological samples essentially combines a sample preparation step that extracts the target compounds from the matrix, with a separation technique that generates the sample composition. Regarding that SCFA are probably the most abundant family present in biological samples from ruminants, GC is the most suitable separation approach to deal with these samples. In contrast to the high number of recent reports about the application of miniaturized extraction techniques to biological samples,^10^ the analytical developments for analyzing biological samples from ruminants are still limited. This is even more critical for methods applied to rumen fluid, which cannot be directly translated from applications focused on human biological samples. Hence, the combination of simple and upscalable sample preparation protocols with GC, a common instrumental analysis present in most animal nutrition laboratories, can provide relevant analytical information about the nutritional and health status of the animal.

Among the classic sample preparation toolbox for GC,^11^ several valid options are available, being solid-phase microextraction (SPME), solid-phase extraction (SPE), or liquid–liquid extraction (LLE) among the most widespread techniques. Considering the predominant volatile character of some potential nutritional biomarkers from ruminants, SPME is a common suitable choice due to the possibility to extract and concentrate the analytes in a single step, associated with the possibility of bypassing the use of organic solvents when thermal desorption^12^ is applied. Nevertheless, the presence of nonvolatile compounds and the complexity of the sample matrix can compromise the performance of the SPME fiber if in contact with the sample^13^ after a limited number of extractions. Considering SPE, it can also be a suitable tool for extracting and concentrating organic molecules with potential nutritional biomarking interest. This is motivated by the high selectivity and retention capacity of the current generation of sorbent materials.^14^ The major weakness of this approach is related to the potential loss of volatile compounds, the difficulty to implement manual protocols in small-medium laboratories without prior experience, as well the low throughput of SPE under manual operation format. Furthermore, both SPE and SPME imply an implementation cost that may create a barrier to newcomers, even in a small-scale format. On the other hand, LLE can be a suitable extraction technique to overcome this barrier. LLE uses regular chemicals (e.g., solvents, salts), supplies (e.g., tubes, centrifuge microtubes), and apparatuses (e.g., vortex, centrifuges) common to analytical and animal nutrition laboratories.^15^ Therefore, the development of miniaturized approaches for LLE can contribute to develop simple and versatile methods for the extraction of biological samples that are compatible with Green Chemistry principles.^16^ Indeed, LLE has been used for extraction of biomolecules in urine^17^ and feces.^18^ Additionally, LLE was already applied to the extraction of metabolites from rumen fluid^19^ prior to LC analysis. In the same work, it was possible to tune the acid–base character of the solvent composition and the salt content to maximize the extraction yields. This resulted in the identification of 614 compounds, a number significantly higher than the one obtained by the other extraction techniques (SPE and its variant QuEChERS) applied to the same samples. Although LLE seems to be a promising technique for simplifying the analysis of biological samples from ruminants, the current state of the art still lacks sample preparation protocols dedicated and optimized to this end.

In order to fill this methodological gap, this work aimed to develop a simple, efficient, and green method based on LLE aided by salting-out for extracting volatile metabolites present in biological samples from ruminants—rumen fluid, urine, feces—that was further combined with GC-FID for multiclass analysis of these compounds.

## Materials and Methods

### Reagents and Solutions

All chemicals were of analytical grade. 1-Hexanol, 1-phenylethanol, 2,3-pentanedione, 2,6-dimetoxyphenol, 2-butanone, 2-heptanone, 2-hexanone, 2-pentanone, 2-phenylethanol, 2-phenylethylacetate, 2-propenal, 3-ethylphenol, 3-hexanone, 3-methyl-2-pentanone, 3-methylbutanal, 3-octanol, 3-octanone, 4-ethylguaiacol, 4-ethylphenol, 4-methyl-2-pentanone, 4-vinylphenol, acetaldehyde, acetic acid, dimethyl sulfide, acetoin, benzaldehyde, butanal, butyl butyrate, butyric acid, caproic acid, decanal, diacetyl, dimethyl disulfide, dimethyl trisulfide, ethyl 2-methylbutyrate, ethylphenylacetate, ethyl butyrate, ethyl hexanoate, ethyl isovalerate, eugenol, furfural, heptanal, hexanal, hexyl ether, isoamyl acetate, isoamyl hexanoate, isobutanal, isobutyl isovalerate, isobutyric acid, isovaleric acid, *m*-cresol, methyl 2-methylbutyrate, methyl propyl disulfide, nona-2,4-dienal, nonanal, octanal, *p*-cresol, pentanal, phenol, propanal, propionic acid, *tert*-butyl methyl sulfide, *trans*-2-decenal, *trans*-2-heptenal, *trans*-2-hexenal, *trans*-2-nonenal, *trans*-2-octenal, *trans*-2-pentenal, valeric acid, and ammonium sulfate were purchased from Sigma-Aldrich (Madrid, Spain). 1-Propanol was purchased from PanReac AppliChem (Barcelona, Spain), methanol, acetone, ethanol, and hydrochloric acid (HCl, 37%, w/w) were supplied from Chem-Lab (Zedelgem, Belgium), 2-methylbutanal was purchased from Alfa Aesar (Haverhill, MA, USA), and 2-propanol from Fisher Scientific (Hampton, NH, USA). The stock standard solutions were prepared in diethyl ether (stabilized with 6 mg L^–1^ BHT; PanReac AppliChem) and stored at −20 °C until further use.

### Samples

All the experimental animal procedures were conducted at the Vairão Agricultural Campus of Abel Salazar Biomedical Sciences Institute, University of Porto (ICBAS-UP; Vila do Conde, Portugal). Cows were handled in strict accordance with good animal practices as defined by the National authority and European Union Directive 2010/63/EU. Experimental animal procedures were approved by the Local Animal Ethics Committee of ICBAS-UP, licensed by the Portuguese Directorate-General of Food and Veterinary Medicine of the Ministry for Agriculture and Sea (permit #0421/000/000/2015), and conducted by trained scientists (FELASA category C). All samples (rumen fluid, urine, and feces) were obtained 2 h after the morning feed from 3 multiparous lactating Holstein cows fitted with a ruminal cannula (10 cm diameter; Bar Diamond Inc., Parma, ID, USA). Cows had access to fresh drinking water *ad libitum* and were fed total mixed rations (TMR) based on maize silage, averaging (dry matter basis) 432 g kg^–1^ neutral detergent fiber, 103 g kg^–1^ crude protein, and 267 g kg^–1^ starch.

Ruminal contents were collected from the four quadrants of the rumen, homogenized, and filtered through four layers of linen-cloth to 50 mL plastic centrifuge tubes. Filtered rumen fluid were centrifuged (10 min, 350*g*, Astor 8 NEW Gerber Centrifuge, Astori Technica, Poncarale, Italy) to remove the suspended particles, and the supernatant transferred to 50 mL plastic centrifuge tubes. The urine samples were collected by stimulation of the vulva to 50 mL centrifuge plastic tubes and acidified to pH ∼ 1–2 with hydrochloric acid (final concentration of ∼2 mol L^–1^).^20^ The feces were collected during defecation or directly from the rectum into plastic bags with hermetic seals. All samples were placed on ice immediately after collection and transported to the laboratory. All samples were kept at −20 °C until further use.

### Extraction Procedure

**Figure 1:**
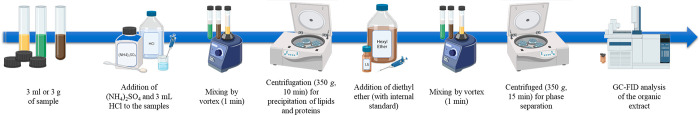
Scheme of the extraction procedure.

Considering the diversity of compounds and the complexity of the biological matrices under study, the development of the LLE protocol comprised three steps: (i) the establishment of the initial conditions for the optimization, (ii) a first-phase optimization based on a full-factorial design of experiments (DoE) to identify the relevant factors of the extraction, and (iii) a second-phase optimization based on a Central Composite Design DoE to define the parameters of the LLE. Experimental setups for the optimization of the LLE conditions using DoE were generated and analyzed using Minitab 14 statistical software (State College, PA, USA). From these preliminary studies, the following extraction procedure was established (Figure 1). In 22 mL centrifuge glass tubes, 3 mL of ruminal fluid sample (3 mL of urine or 3 g for feces) were mixed with 3 mL of 5 mol L^–1^ hydrochloric acid (7 mol L^–1^ for urine or 6 mol L^–1^ for feces) and solid of ammonium sulfate at a final concentration of 1 mol L^–1^ in the sample (6 mol L^–1^ for urine or 0.04 mol L^–1^ for feces). The mixture was vortexed for 1 min and centrifuged (Astor 8 NEW Gerber Centrifuge) at 350*g* for 10 min at room temperature (∼23 °C) to precipitate proteins and lipids. Then, 2 mL of diethyl ether, containing 5.15 × 10^–2^ mmol L^–1^ of the internal standard (hexyl ether) were added to the tubes, vortexed for 1 min and centrifuged for 15 min at 350*g*. Finally, the organic phase was collected, placed in a 1.5 mL GC glass vial, and immediately analyzed by GC-FID according to the procedure described in the section GC-FID Conditions. The samples were analyzed in triplicate.

#### GC-FID Conditions

The chromatographic analyses were performed by gas chromatography with flame ionization detector (GC-FID), using a GC-2010 Plus (Shimadzu Corporation, Kyoto, Japan) equipped with an DB-Wax UI (30 m × 0.25 mm, 0.25 μm) column (Agilent Technologies, Santa Clara, CA, USA). The temperature was held at 250 °C in the injector and at 260 °C in the detector. The temperature gradient in the oven started at 40 °C, increased to 60 °C at a rate of 2 °C min^–1^, then increased to 200 °C at a rate of 3 °C min^–1^ (holding 2 min) and to 230 °C at a rate of 40 °C min^–1^ (holding 4 min). The carrier gas was helium at a flow rate of 1.05 mL min^–1^. The injection volume was 1 μL using a split ratio of 1:20 (helium at a flow rate of 25 mL min^–1^). The compounds were identified by comparing the retention time of the peaks with those of standards and quantified by external calibration with internal standard (hexyl ether at a concentration of 5.15 × 10^–2^ mmol L^–1^). The calibration curves were constructed by the analysis of standards of the volatile compounds prepared in diethyl ether in the range 4 × 10^–3^ to 0.40 mmol L^–1^, except for SCFA (2–40 mmol L^–1^). The recovery assays were performed by spiking the samples with standard solutions at 3 concentration levels, as described in Table S1. Intraday precision (expressed as relative standard deviation, RSD) was evaluated by the analysis of 5 replicates of the same sample in the same day.

## Results and Discussion

### Rationale and Requirements of the Analytical Method

The monitoring of rumen function is usually assessed by the direct analysis of rumen fluid, which involves invasive procedures, being thus relevant to identify biomarkers in biological samples provided by noninvasive collection. For that purpose, a method was developed and optimized to target multiple classes of volatile metabolites formed during rumen fermentation: aldehydes, ketones, phenols, alcohols, sulfides, and esters. Considering the high range of polarity and volatility of the targeted compounds, the complexity of the different biological matrices (rumen fluid, urine, and feces), and the need for a method that followed the Green Chemistry principles,^16^ we identified miniaturized LLE as a suitable solution to deal with these samples. In order to facilitate the implementation of the method, we chose GC-FID for identification and quantification of the compounds present in each sample.

**Figure 2:**
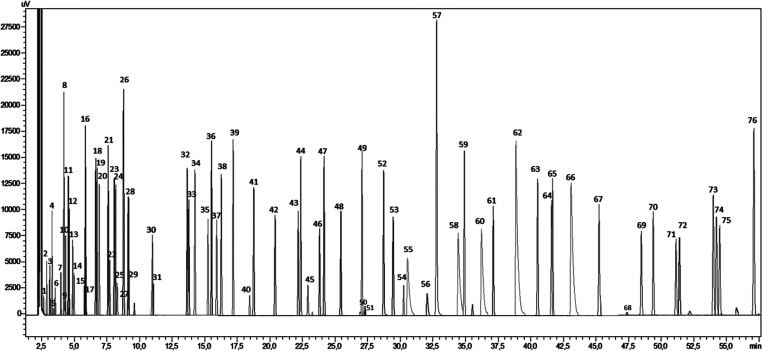
Characteristic GC-FID chromatogram of a standard solution (0.25 mmol L^–1^) containing the analyzed compounds. (1) Acetaldehyde; (2) dimethyl sulfide; (3) propanal; (4) isobutanal; (5) acetone; (6) 2-propenal; (7) butanal; (8) *tert*-butyl methyl sulfide; (9) methanol; (10) 2-butanone; (11) 2-methylbutanal; (12) 3-methylbutanal; (13) 2-propanol; (14) ethanol; (15) 2,3-butanedione; (16) 2-pentanone; (17) pentanal; (18) 4-methyl-2-pentanone; (19) methyl 2-methylbutyrate; (20) 3-methyl-2-pentanone; (21) ethyl butyrate; (22) 1-propanol; (23) 3-hexanone; (24) ethyl 2-methylbutyrate; (25) 2,3-pentanedione; (26) ethyl isovalerate; (27) dimethyl disulfide; (28) 2-hexanone; (29) hexanal; (30) isoamyl acetate; (31) *trans*-2-pentenal; (32) 2-heptanone; (33); heptanal; (34) isobutyl isovalerate; (35) *trans*-2-hexenal; (36) butyl butyrate; (37) methyl propyl disulfide; (38) ethyl hexanoate; (39) 3-octanone; (40) 3-hidroxybutanone; (41) octanal; (42) *trans*-2-heptenal; (43) 1-hexanol; (44) hexyl ether (internal standard); (45) dimethyl trisulfide; (46) nonanal; (47) 3-octanol; (48) *trans*-2-octenal; (49) acetic acid; (50) isoamyl hexanoate; (51) furfural; (52) decanal; (53) benzaldehyde; (54) *trans*-2-nonenal; (55) propionic acid; (56) isobutyric acid; (57) 4-vinylphenol; (58) butyric acid; (59) *trans*-2-decenal; (60) isovaleric acid; (61) nona-2,4-dienal; (62) valeric acid; (63) ethylphenylacetate; (64) 1-phenylethanol; (65) 2-phenylethylacetate; (66) caproic acid; (67) BHT (diethyl ether stabilizer); (68) 2-phenylethanol; (69) phenol; (70) 4-ethylguaiacol; (71) 4- methylphenol; (72) 3-methylphenol; (73) eugenol; (74) 4-ethylphenol; (75) 3-ethylphenol; (76) 2,6-dimetoxyphenol.

The establishment of the chromatographic separation was based on a previous study for the analysis of multiclass organic compounds using a polar GC column.^21^ Standard solutions of the volatile compounds were prepared by dilution or dissolution of the commercial reagents described in the section Reagents and Solutions in diethyl ether and injected individually in the GC-FID system to establish the chromatographic conditions for optimal separation of the peaks and their retention times. A chromatogram of a standard solution containing the 75 tested compounds is shown in Figure 2.

### Development of the LLE Protocol for the Biological Samples

The feces and ruminal fluid samples presented a challenge due to their heterogeneity; an amount of 3.00 mL of rumen fluid and 3.00 g of feces were selected for this study. Urine samples tend to be less heterogeneous, but for sake of simplicity during the development of the extraction protocol, we selected the same volume of 3.00 mL applied to rumen fluid.

In the first attempts made in the laboratory to develop the methodology, it was observed that the pH of the sample affected the extraction of compounds; the extraction of the samples only with diethyl ether was insufficient to recover the target metabolites from the matrices. The addition of an acid (hydrochloric acid, phosphoric acid and trichloroacetic acid) was tested to promote the extraction. The extraction with trichloroacetic acid resulted in a poor liquid phase separation, which caused the appearance of a wide peak in the GC-FID chromatogram (Figure S1). Phosphoric acid and hydrochloric acid had similar performance in both liquid phases separation and in the chromatographic separation, although slightly higher peak areas were obtained using hydrochloric acid (Figure S1). For that reason and according to the information found elsewhere,^22^ hydrochloric acid was chosen for samples’ acidification. The influence of the acid was more prominent on the extraction of the acidic species (the SCFA), one of the most important groups of volatile metabolites in ruminant biological samples, since at low pH values (pH < 3) these compounds are in their unprotonated form, enabling its extraction from the sample to the organic phase. Another factor that influenced the efficiency of the extraction was the addition of a salt. The salting-out effect is recognized as an enhancer of the efficiency of both the extraction and the phase separation, with positive results on the precipitation of proteins.^23,24^ The selected salt was ammonium sulfate, (NH_4_)_2_SO_4_, due to its recognized advantages on the protein precipitation and phase separation, without altering the samples’ pH.^23^

**Figure 3:**
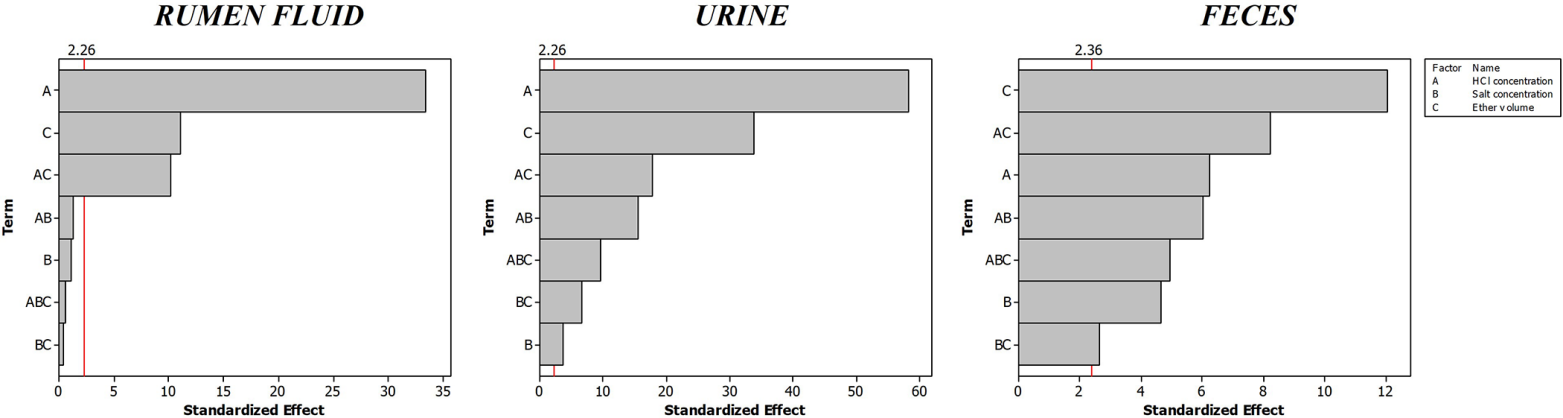
Pareto charts resulting of the full factorial design to the optimization of the liquid–liquid extraction of rumen fluid, urine, and feces samples. A: HCl concentration; B: salt concentration; C: diethyl ether volume.

Considering the above-mentioned result of the preliminary studies of the LLE, we performed a full factorial experimental design to evaluate the effect of the experimental factors (HCl concentration, (NH_4_)_2_SO_4_ concentration, and diethyl ether volume) on the analytical response; the assays were performed in duplicate. The analytical response factor was defined as the total area of the identified peaks. The full factorial design comprised two levels (−1, +1; in duplicate) for each factor (HCl: 0–6 mol L^–1^ for rumen fluid and urine and 0.5–6 mol L^–1^ for feces; (NH_4_)_2_SO_4_: 1–5 mol L^–1^; diethyl ether volume: 2–4 mL) and 2 center points (0; in triplicate), corresponding to a total of 18 experimental runs for each matrix. The analysis of the standardized pareto charts (Figure 3) showed that all the factors and interactions between them significantly (*p* < 0.05) affected the extraction of the compounds from urine and feces samples (Figure 3B and 3C). For rumen fluid samples (Figure 3A), only the concentration of HCl, the volume of diethyl ether, and the interaction of these two factors significantly (*p* < 0.05) affected the extraction efficiency. For the subsequent work, the salt concentration was kept at the minimum value tested (1 mol L^–1^) for rumen samples.

Considering the results obtained from the full factorial design, a central composite design was subsequently performed to optimize the LLE protocol. For rumen fluid samples, the design comprised 4 cube points, 2 center points, and 4 axial points, in duplicate, in a total of 20 experimental runs. For urine and feces samples, the design consisted of 8 cube points, 2 center points, and 6 axial points, in duplicate, in a total of 32 experimental runs. The studied ranges and the optimized values obtained to achieve the maximum total area of the chromatographic peaks for each factor are presented in Table 1.

**Table 1:** Studied Ranges and Optimized Values for the Studied Factors in the Central Composite Design Experiments for the Three Studied Samples

		ether volume (mL)	HCl concentration (mol L^–1^)	salt concentration (mol L^–1^)
rumen fluid	studied range	1.80–4.50	0.1–7.5	–
	optimized values	2.00	5.0	–a
urine	studied range	1.00–3.50	0.5–7.4	1.0–6.0
	optimized values	2.00	7.0	6.0
feces	studied range	1.30–4.70	0.1–7.5	0.04–6.3
	optimized values	2.00	6.0	0.04

a For the central composite design, the salt concentration for rumen fluid samples extraction was kept at 1 mol L^–1^ according to the preliminary studies as described in the text.

**Figure 4:**
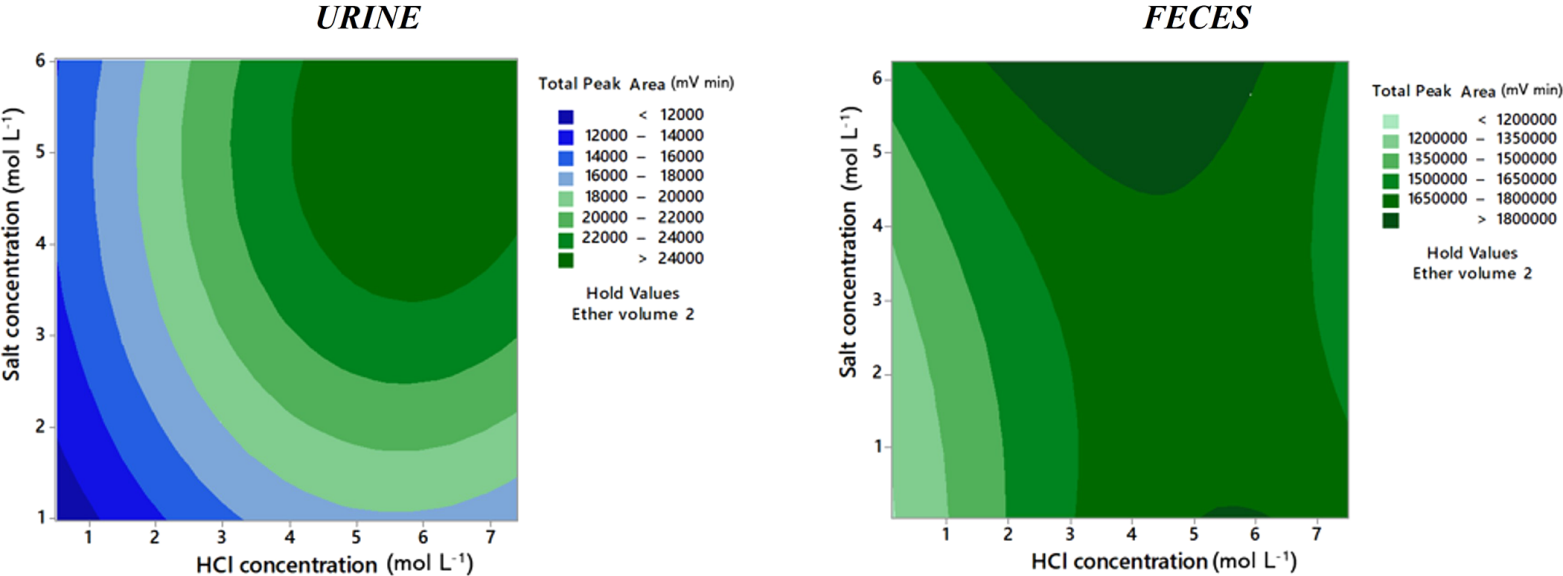
Contour plots of salt concentrations versus HCl concentration resulting from the central composite design for urine and feces samples.

From the experiments, it was observed that using ether volumes below 2 mL it was difficult to collect a clear and sufficient volume of organic extract for GC analysis in some samples, especially for rumen fluid and feces samples. For that reason, it was decided to adopt 2 mL as the ether volume. The HCl and salt concentrations were selected according to the results of the central composite design experiments. The results are shown in the contour plots presented in Figure 4. In urine samples, it was verified that higher extraction efficiency was obtained for the highest values of concentrations tested; 7.0 mol L^–1^ and 6 mol L^–1^ of HCl and ammonium sulfate, respectively, were selected as optimal conditions. For feces, it was observed that maximum peak areas were obtained for the concentration of ammonium sulfate above 4.5 mol L^–1^ or below 0.1 mol L^–1^, while for HCl concentration optimal values were in the range 2–6 mol L^–1^. Considering that high salt concentrations caused some difficulties in the extraction of certain feces samples, the conditions 6.0 mol L^–1^ of HCl and 0.04 mol L^–1^ of ammonium sulfate were chosen.

After the definition of the optimum values for each factor (Table 1), samples with similar characteristics to the ones used for the optimization process were analyzed, and the total area of their chromatograms was compared with the values predicted by the optimization model. It was observed that the total area of the chromatograms of these samples was 92, 94, and 85% of the areas predicted by the optimization model for rumen fluid, urine, and feces, respectively.

### Analytical Features of the Methodology

The proposed analytical methodology was characterized in terms of linearity, detection, and quantification limits for the compounds detected in the studied samples. To evaluate the linearity range of the methodology, calibration curves of the standards were prepared by dilution of the stock standard solutions in ultrapure water (in the range 4 × 10^–3^ to 0.40 mmol L^–1^, except for SCFA: 2–40 mmol L^–1^), and submitted to the extraction protocols for each sample as described in the Extraction Procedure section. Linear regressions of the peak area of the analyte/peak area of the internal standard vs concentration of the analyte were calculated. Linearity of the calibration curves was assessed by the coefficient of determination (*r*^2^), being the obtained values above 0.991 (Table 2). The detection and quantification limits (LOD and LOQ, respectively) were calculated as 3 and 10 times the standard deviation of the slope of the linear regression, respectively.^25,26^ The LOD and LOQ for the studied compounds are presented in Table 2. Intraday precision values (Table S1) varied between 1% and 24% for the spiking of the samples with standard solutions at 3 concentration levels. Recoveries (Table S1) varied in the following ranges: rumen fluid, 75–113%, urine, 68–115%, and feces, 70–108%.

**Table 2:** Coefficient of Determination (*r*^2^), Limits of Detection (LOD) and Quantification (LOQ) of the Methodology

			rumen fluid			urine			feces		
peak no.^a^	compounds	*t*_R_ (min)	*r*^2^	LOD (μmol L^–1^)	LOQ (μmol L^–1^)	*r*^2^	LOD (μmol L^–1^)	LOQ (μmol L^–1^)	*r*^2^	LOD (μmol L^–1^)	LOQ (μmol L^–1^)
Short-Chain Fatty Acids											
49	acetic acid	26.9	0.994	3.88 × 10^3^	1.29 × 10^4^	1.000	3.22 × 10^2^	1.07 × 10^3^	1.000	4.20 × 10^2^	1.40 × 10^3^
55	propionic acid	30.82	0.996	3.03 × 10^3^	1.01 × 10^4^	1.000	8.98 × 10^2^	2.99 × 10^3^	1.000	1.58 × 10^2^	5.26 × 10^2^
56	isobutyric acid	32.29	0.999	1.23 × 10^2^	4.10 × 10^2^	0.999	38.16	1.27 × 10^2^	1.000	18.88	62.93
58	butyric acid	34.68	0.997	2.47 × 10^3^	8.24 × 10^3^	1.000	2.53 × 10^2^	8.45 × 10^2^	1.000	3.42 × 10^2^	1.14 × 10^3^
60	isovaleric acid	36.58	1.000	1.88 × 10^2^	6.26 × 10^2^	0.998	6.45 × 10^2^	2.15 × 10^3^	1.000	76.01	2.53 × 10^2^
62	valeric acid	39.22	1.000	51.50	1.72 × 10^2^	1.000	2.27 × 10^2^	7.57 × 10^2^	1.000	1.81 × 10^2^	6.04 × 10^2^
66	caproic acid	43.47	0.999	47.14	1.57 × 10^2^	0.999	1.05 × 10^2^	3.50 × 10^2^	1.000	47.64	1.59 × 10^2^
Aldehydes											
3	propanal	3.13	1.000	0.25	0.84	1.000	0.24	0.79	1.000	0.18	0.61
4	isobutanal	3.31	1.000	0.82	2.75	1.000	1.26	4.20	1.000	0.20	0.66
7	butanal	3.98	0.996	4.52	15.07	0.997	4.24	14.15	0.999	1.91	6.35
54	*trans*-2-nonenal	30.27	1.000	1.83	6.10	1.000	0.26	0.85	0.999	2.39	7.96
61	nona-2,4-dienal	37.20	1.000	0.63	2.11	0.993	5.99	19.96	1.000	0.29	0.96
Alcohols											
13	isopropanol (2-propanol)	4.89	0.991	7.61	25.37	0.996	4.56	15.20	1.000	1.50	4.99
64	1-phenylethanol	41.61	0.999	1.00	3.34	0.992	5.83	19.42	0.999	2.28	7.62
Ketones											
5	acetone	3.40	1.000	0.85	2.83	1.000	0.87	2.90	1.000	1.12	3.75
10	2-butanone	4.43	1.000	0.23	0.77	0.999	2.03	6.78	1.000	0.09	0.29
Esters											
19	methyl 2-methylbutyrate	6.92	0.999	1.66	5.52	0.992	7.82	26.08	1.000	1.21	4.02
63	ethyl phenylacetate	41.61	1.000	0.21	0.69	0.998	1.23	4.09	1.000	0.47	1.55
Phenols											
71	4-methylphenol (*p*-cresol)	51.63	1.000	1.11	3.70	1.000	0.79	2.62	1.000	0.27	0.90
72	3-methylphenol (*m*-cresol)	51.90	1.000	1.11	3.70	1.000	0.79	2.62	1.000	0.27	0.90
74	4-ethylphenol	54.26	0.998	3.23	10.76	1.000	0.27	0.90	1.000	0.27	0.90
75	3-ethylphenol	54.92	0.992	5.83	19.42	1.000	0.27	0.90	1.000	1.12	3.73
Sulfides											
2	dimethyl sulfide	2.89	1.000	0.12	0.41	0.998	3.21	10.70	1.000	0.11	0.38
8	*tert*-butyl methyl sulfide	4.21	1.000	0.06	0.21	1.000	0.27	0.89	0.997	2.35	7.84

a Peak numbers correspond to those presented in Figure 2.

### Application of Methodology in the Biological Samples

**Figure 5:**
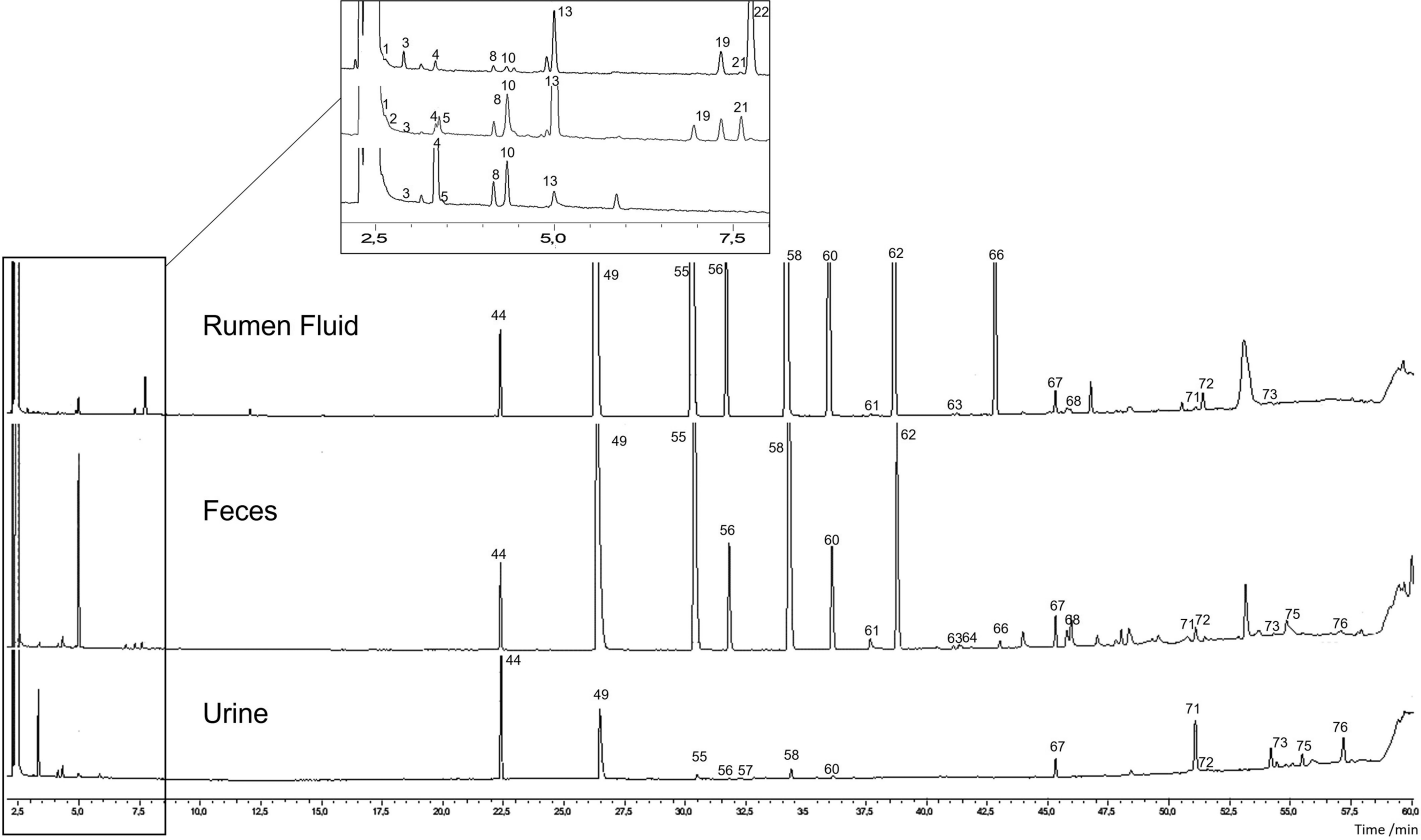
Typical GC-FID chromatograms of extracts of rumen fluid, feces, and urine samples obtained by the developed methodology. Peak numbers correspond to the compounds described in Figure 1.

The optimized methodology was applied to samples of rumen fluid, urine, and feces collected from three cows according to the protocol described in the section Samples. Typical chromatograms of the extracts of the samples are presented in Figure 5.

Average results from the three cows are present in Table 3. The studied sample matrices presented different compounds, most of them known to be related with characteristics of the ingested diet, rumen fermentation, and animal metabolism.

**Table 3:** Concentration (± Standard Error of the Mean) of the Studied Compounds in Samples of Rumen Content, Urine, and Feces Collected from Dairy Cows (A1, A2, and A3)

	rumen fluid (μmol L^–1^)^a^			urine (μmol L^–1^)			feces (μmol kg^–1^)		
compounds	A1	A2	A3	A1	A2	A3	A1	A2	A3
Short-Chain Fatty Acids									
acetic acid	6.33 × 10^4^ ± 1.54 × 10^3^ a	8.81 × 10^4^ ± 3.48 × 10^3^ b	8.36 × 10^4^ ± 9.74 × 10^3^ a,b	1.32 × 10^3^ ± 19.71 a	6.31 × 10^3^ ± 390 c	3.79 × 10^3^ ± 117.17 b	3.97 × 10^4^ ± 2.09 × 10^3^ a	55964.26 ± 3718.26 b	7.54 × 10^4^ ± 3.76 × 10^3^ c
propionic acid	1.99 × 10^4^ ± 4.73 × 10^2^ a	3.15 × 10^4^ ± 1.31 × 10^3^ b	2.49 × 10^4^ ± 3.25 × 10^3^ a,b	ND	ND	ND	7.08 × 10^3^ ± 4.31 × 10^2^ a	1.06 × 10^4^ ± 7.65 × 10^3^ b	1.83 × 10^4^ ± 9.60 × 10^3^ c
butyric acid	1.15 × 10^4^ ± 2.96 × 10^2^ a	1.77 × 10^4^ ± 7.25 × 10^2^ b	1.67 × 10^4^ ± 2.23 × 10^3^ b	ND	ND	ND	2.02 × 10^3^ ± 2.02 × 10^2^ a	2.58 × 10^3^ ± 2.85 × 10^2^ a	6.54 × 10^3^ ± 3.73 × 10^2^ b
isobutyric acid	6.49 × 10^2^ ± 18.48 a	8.83 × 10^2^ ± 38.08 a,b	1.023 × 10^3^ ± 1.45 × 10^2^ b	ND	ND	ND	116.38 ± 5.62 a	368.32 ± 28.66 b	561.17 ± 25.06 c
valeric acid	8.93 × 10^2^ ± 21.93 a	1.50 × 10^3^ ± 59.32 b	1.31 × 10^3^ ± 1.66 × 10^2^ b	ND	ND	ND	ND	NQ	679.82 ± 40.73 a
isovaleric acid	6.40 × 10^2^ ± 6.31 a	1.18 × 10^3^ ± 51.64 a,b	1.40 × 10^3^ ± 1.96 × 10^2^ b	ND	ND	ND	NQ	315.45 ± 13.76 a	391.05 ± 11.45 b
caproic acid	4.86 × 10^2^ ± 12.78 a	7.34 × 10^2^ ± 33.85 b	5.98 × 10^2^ ± 83.25 a,b	ND	ND	ND	ND	ND	ND
Aldehydes									
propanal	NQ	7.00 ± 0.3 a	6.04 ± 0.93 a	2.60 ± 0.25 a	3.57 ± 0.1 b	ND	ND	0.82 ± 0.18 a	1.36 ± 0.18 b
isobutanal	3.05 ± 0.06 a	3.33 ± 0.13 a	6.67 ± 1.12 a	ND	106.89 ± 7.65 b	25.49 ± 2.86 c	ND	ND	ND
butanal	ND	ND	ND	ND	ND	ND	ND	13.68 ± 0.99 a	ND
*trans*-2-nonenal	ND	ND	ND	ND	3.19 ± 0.42 a	2.79 ± 0.18 a	ND	ND	ND
nona-2,4-dienal	ND	ND	4.02 ± 1.01 a	ND	ND	ND	3.52 ± 0.29 a	9.59 ± 1.11 b	7.48 ± 0.28 b
Alcohols									
2-propanol	34.82 ± 3.06 a	429.76 ± 19.49 b	41.16 ± 4.32 a	24.30 ± 1.55 a	30.61 ± 2.68 a	28.87 ± 1.04 a	6153.24 ± 457.67 a	7667.31 ± 1209.39 a	5596.21 ± 1228.87 a
1-phenylethanol	4.26 ± 0.15 a	6.74 ± 0.28 a	7.11 ± 1.94 a	ND	ND	ND	9 ± 0.41 a	20.64 ± 1.87 b	30.57 ± 1.16 c
Ketones									
acetone	ND	ND	ND	ND	ND	ND	53.15 ± 3.08 a	62.67 ± 3.01 a	67.31 ± 7.27 a
2-butanone	2.89 ± 0.26 a	2.95 ± 0.25 a	3.03 ± 0.54 a	ND	ND	ND	4.51 ± 0.5 a	7.97 ± 0.89 b	11.34 ± 0.94 c
Esters									
methyl 2-methylbutyrate	ND	ND	ND	ND	ND	ND	4.25 ± 0.18 a	5.2 ± 0.19 a,b	5.49 ± 0.39 b
ethylphenylacetate	ND	ND	1.73 ± 0.55 a	ND	ND	ND	1.48 ± 0.01 a	2.13 ± 0.11 b	3.36 ± 0.1 c
Phenols									
4-methylphenol (*p*-cresol)	9.58 ± 0.3 b	4.98 ± 0.21 a	12.59 ± 1.78 b	11.22 ± 0.76 a	78.49 ± 9.12 b	18.57 ± 1.33 a	ND	13.94 ± 1.58 a	NQ
3-methylphenol (*m*-cresol)	ND	19.31 ± 0.96 a	18.05 ± 2.7 a	ND	4.85 ± 0.96 a	5.58 ± 0.31 a	2.3 ± 0.13 a	2.52 ± 0.29 a	4.98 ± 0.3 b
3-ethylphenol	ND	ND	ND	NQ	1.68 ± 0.23 a	1.26 ± 0.15 a	11.18 ± 0.41 b	6.89 ± 0.7 a	7.94 ± 0.65 a
4-ethylphenol	ND	ND	ND	ND	1.24 ± 0.27 a	1.34 ± 0.3 a	ND	ND	ND
Sulfides									
dimethyl sulfide	2.62 ± 0.04 a	12.98 ± 0.37 b	2.93 ± 0.24 a	ND	ND	ND	ND	1.46 ± 0.17 a	ND
*tert*-butyl methyl sulfide	0.41 ± 0.05 a	0.51 ± 0.08 a	3.52 ± 0.63 b	ND	6.17 ± 0.5 b	2.2 ± 0.12 a	7.63 ± 0.2 a	10.88 ± 0.61 b	9.42 ± 0.34 a,b

a Different letters indicate significant (*p* < 0.05) differences between concentration values within samples. ND: not detected; NQ: not quantified.

#### Rumen Fluid

From the rumen microbial fermentation of carbohydrates, a large amount of SCFA, mainly acetic, propionic, and butyric acids, are produced in the rumen, their proportions depending on the type of carbohydrate fermented; SCFA are absorbed through the rumen wall and mainly used as energy source by the host,^27^ representing nearly 70% of the metabolic energy utilized by the animal.^2^ After passing across the rumen wall, acetic acid is a primary precursor of short- and medium-chain fatty acids, propionic acid is a primary glycogenic precursor in the liver, and butyric acid is a lipogenic precursor of longer-chain fatty acids.^2,28^ Smaller quantities of other SCFA can be also formed in the rumen by deamination of amino acids, as isobutyric, isovaleric, and valeric acids.^28^ Acetic acid was the SCFA found at higher concentration (6.33 × 10^4^ to 8.81 × 10^4^ μmol L^–1^), followed by propionic acid (1.99 × 10^4^ to 3.15 × 10^4^ μmol L^–1^) and butyric acid (1.15 × 10^4^ to 1.77 × 10^4^ μmol L^–1^), whereas the concentrations of isobutyric, valeric, isovaleric, and caproic acids in the rumen fluid varied between 4.86 × 10^2^ and 1.50 × 10^3^ μmol L^–1^ (Table 3).

Propanal and isobutanal, short-chain aldehydes formed as a byproduct of carbohydrates fermentation,^29^ were measured in the analyzed samples in the ranges 6.04–7.00 μmol L^–1^ and 3.05–6.67 μmol L^–1^, respectively (Table 3). Nona-2,4-dienal, a long-chain aldehyde, was measured in the rumen fluid samples (4.05 μmol L^–1^, Table 3). This aldehyde is found in some feed ingredients such as soybean and comprise a flavoring compound used in animal feed (EC 1831/2003).^30^

The synthesis of alcohols in the rumen occurs by fungi and bacteria activity.^31^ As alcohol removal from the rumen follows microbial metabolism,^32^ the balance between the inputs and outputs is difficult to study and predict. The synthesis of isopropanol (2-propanol) has been attributed to the microbial reduction of acetone or from decarboxylation of β-hydroxybutyrate,^33^ thus being associated with ketosis at levels up to 3.2 × 10^3^ μmol L^–1^.^34,35^ In the present study, isopropanol and 1-phenylethanol were determined in rumen fluid at 34.82–429.76 μmol L^–1^, and 4.26–7.11 μmol L^–1^, respectively (Table 3). These levels of isopropanol are within the range determined by Sato and Shiogama^36^ in healthy animals. The presence of 1-phenylethanol was measured in milk^37^ but its content in the rumen is undocumented, to the best of our knowledge. Its synthesis is related to the metabolism of the amino acid l-phenylalanine and can be a marker of protein digestion.^37^

The formation of ketones can be attributed to the microbial fermentation of carbohydrates.^38^ In the present study, only 2-butanone was detected in the samples of rumen fluid (2.89–3.03 μmol L^–1^, Table 3).

Esters result from the activity of gut wall and bacterial esterases on SCFA,^39^ being ethylphenylacetate (2-phenylbutyrate), formed through the butanoate metabolism,^40^ found in rumen fluid (1.73 μmol L^–1^, Table 3).

In the present work, only two phenols were quantified in rumen fluid: the highest content being 3-methylphenol (*m*-cresol, 18.05–19.31 μmol L^–1^), followed by 4-methylphenol (*p*-cresol, 4.98–12.59 μmol L^–1^). The formation of phenols in the rumen is unclear, as it has different possible sources: (i) conversion of lignin and diterpenes from feed; (ii) as products of the protein digestion through the degradation of tryptophan and tyrosine to phenols such as 4-methylphenol and 4-ethylphenol; or (iii) the microbial decarboxylation of organic acids.^41^ Fraser et al.^42^ studied the formation of phenols (4-methylphenol, 3-methylphenol, and phenol) in the rumen of sheep grazing pastures of different composition and observed that the kinetics of formation and the profile of these compounds in the rumen was different, concluding that the time of collection of the rumen fluid sample may impact the measured phenols’ concentration. These authors observed that approximately 2 h after feeding the concentration of 4-methylphenol and phenol reached their maximum, while 3-methylphenol maximum concentration was achieved 6 h after feeding, independently of the feed composition. O’Callaghan et al.^43^ observed that the formation of 4-methylphenol was higher in animals fed pasture (perennial ryegrass and perennial ryegrass and white clover) than those fed a TMR (65.95 and 85.18 μmol L^–1^ vs 58.38 μmol L^–1^). The higher formation of 4-methylphenol in the rumen in animals fed fresh pastures has been also associated with β-carotene degradation. The ensiling process and the production of concentrates is responsible for the degradation of many carotenoids, being the reason for the low β-carotene levels on TMR diets.^44^ This fact can explain the lower *p*-cresol levels determined in the present work, in which cows were fed TMR.

Sulfides have been reported as important components of rumen headspace and, consequently, of the cows’ breath.^45^ Their formation in the rumen is related to the digestion of proteins, particularly the breakdown of sulfur-containing amino acids such as methionine.^46^ Due to their high volatility, sulfides are important components of the rumen headspace gas (up to 200 mg/kg^45^), the concentration on the liquid phase (rumen fluid) being much lower. Dimethyl sulfide was the main sulfide found in our study (2.62–12.98 μmol L^–1^, Table 3). The content of this sulfide in the rumen headspace is highly dependent on the time of sampling, as a higher production occurs immediately after feeding.^45^ Also, *tert*-butyl methyl sulfide was determined in the range 0.41–3.52 μmol L^–1^ (Table 3); yet no information related to the presence of this sulfurous compound in the rumen was found in the literature.

#### Urine

Most of the metabolic waste, namely toxic compounds formed during metabolism, are excreted in the urine. Therefore, the SCFA, aldehydes, and alcohols formed as byproducts of the carbohydrate, protein, and lipid degradation were determined in the urine. In our study, acetic acid was the only SCFA determined in the urine samples (1.32 × 10^3^ to 6.31 × 10^3^ μmol L^–1^, Table 3). Propanal (2.60–3.57 μmol L^–1^), isobutanal (25.49–106.89 μmol L^–1^), and *trans*-2-nonenal (2.79–3.19 μmol L^–1^) were also measured in urine samples (Table 3). To the best of our knowledge, their presence in urine is undocumented. However, since their formation in biological systems is associated with the oxidation of fatty acids,^47^ we hypothesized that their formation in urine followed that route, although this hypothesis needs to be confirmed in further studies. The alcohol 2-propanol was found in the range 24.30–30.61 μmol L^–1^ (Table 3), but the information about the presence of this compound in urine is absent in the literature.

Due to the toxicity of phenols, the detoxification occurs in the liver by the formation of sulfate or glucuronide conjugated forms of phenols for their excretion in the urine. For this reason, the majority (more than 94%) of phenols in urine is present in the conjugated form,^48^ and result from the tyrosine metabolism, being excreted typically in urine rather than in feces.^49^ In the present study we only determined the free phenol content, as no hydrolysis was performed before the analysis. The main phenol in urine was 4-methylphenol (*p*-cresol) followed by 3-methylphenol (*m*-cresol), as also observed by other authors;^48,50^ concentrations varying between 11.22 and 78.49 μmol L^–1^ for 4-methylphenol, and 4.85–5.58 μmol L^–1^ for 3-methylphenol (Table 3). The concentrations of both phenols were higher than of those determined by Lane and Fraser^48^ in the urine of cows fed ryegrass/clover and maize based diets (11.4–15.6 and 0.2–2.7 μmol L^–1^, respectively). *P*-cresol is known to be a product of the microbial metabolism of tyrosine.^51^ The origin of 3-methylphenol (*m*-cresol) is unclear, but its formation showed to be higher in animals fed pasture.^48^ The concentration of *m*-cresol in ruminants’ urine is very low compared to *p*-cresol, as described in sheep urine;^50^*m*-cresol was between 0.3% and 4.0% of the combined amount of *m*- and *p*-cresol. Moreover, some chromatographic methods are unable to separate the signals of *m*- and *p*-cresol.^50^ These facts can partially justify the lack of information about the presence of this compound in ruminants’ urine.

The phenol 4-ethylphenol, also formed by the tyrosine metabolism,^48^ was determined at levels in the range 1.24–1.34 μmol L^–1^ (Table 3). These values were lower than those reported by Lane and Fraser^48^ (4.34 μmol L^–1^) in the urine of cattle fed pasture. Suemitsu et al.^52^ reported 4-ethylphenol as one of the more abundant phenols present in cow’s urine. Another phenol, 3-ethylphenol, was present in urine samples (1.26–1.68 μmol L^–1^, Table 3) at concentrations above those reported by other authors.^48^

The only sulfurous compound determined in urine was *tert*-butyl methyl sulfide (2.20–6.17 μmol L^–1^, Table 3). Yet, no information on the presence of this compound in cows’ urine was found in the literature.

#### Feces

The profile of SCFA present in feces can provide insights on the health status of the animal.^39^ The most abundant SCFA present in feces were acetic, propionic, and butyric acids at concentrations in the range 2.02 × 10^3^ to 7.54 × 10^4^ μmol kg^–1^ (Table 3). The SCFA found in feces originates mainly from the microbial fermentation in the large intestine, in particular of undigested protein and fiber;^53^ thus, their profile depends on undigested feed composition, colon microbiota population profile, and on the intestinal transit time.^29^

During the SCFA microbial biosynthesis in the intestine, other minor compounds can be formed as reaction byproducts, including aldehydes, alcohols, ketones and esters, among others.^38^ Propanal and butanal were the only saturated aldehydes determined in the analyzed feces samples (0.82–1.36 μmol kg^–1^ and 13.68 μmol kg^–1^, respectively, Table 3); these compounds are byproducts of the synthesis of the corresponding SCFA, propionic and butyric acids, respectively. The only unsaturated aldehyde quantified in feces was nona-2,4-dienal (3.52–9.59 μmol kg^–1^, Table 3).

As verified by other authors,^36^ alcohols were determined in feces at higher concentrations than in rumen fluid: 2-propanol varied between 5.60 × 10^3^ and 7.67 × 10^3^ μmol kg^–1^, and 1-phenylethanol between 9.00 and 30.57 μmol kg^–1^ (Table 3). Although most alcohols in feces can result from the reduction of aldehydes formed during SCFA synthesis,^38^ 2-propanol is assumed to result from the hydrogenation of acetone by the colonic microbiota, as observed in the rumen,^35^ and to be related to the fiber digestion in the colon.^36^ Aromatic compounds, such as 1-phenylethanol (detected in our samples in the range 4.26–7.11 μmol L^–1^) are originated by bacteria, as part of the shikimate pathway or by degradation of aromatic amino acids l-phenylalanine or l-tyrosine.^38^

Ketones are formed in the lower gut from bacterial conversion of fatty acids,^38,46,54^ but can be also generated endogenously from the lipid and carbohydrate metabolism,^39^ or by peroxidation of long chain fatty acids.^46^ Acetone, and its metabolite 2-propanol, have been detected at increased levels on ketotic cows.^34,35^ In our study, acetone and 2-butanone were determined in the fecal samples of healthy cows as reported by other authors.^36,55^

Esters methyl 2-methylbutyrate and ethylphenylacetate were determined at levels 4.25–5.49 μmol kg^–1^, and 1.48–3.36 μmol kg^–1^ (Table 3). These esters result from the activity of gut wall and bacterial esterases on SCFA.^39^

The majority of phenols formed in the rumen are excreted in urine.^56^ Therefore, phenols detected in the feces are suggested to be originated from fermentation of undigested protein^49^ through the microbial population in the large intestine, which decomposes the aromatic amino acid l-tyrosine into phenolic compounds.^53^ The phenols detected in feces were 4-methylphenol (13.94 μmol kg^–1^), 3-methylphenol (2.30–4.98 μmol kg^–1^), and 3-ethylphenol (6.89–11.18 μmol kg^–1^). No information was found in the literature for the presence of these phenols in ruminant feces; yet their presence can be suggested to be related to phytochemicals from the diet, as in the case of urine.^57^

Sulfurous compounds dimethyl sulfide and *tert*-butyl methyl sulfide were determined in the feces samples at concentrations of 1.46 μmol kg^–1^, 7.63–10.88 μmol kg^–1^, respectively (Table 3). Sulfides are compounds generally found in ruminants’ feces resulting from the microbial conversion of sulfur-containing amino acids.^46^ The relevance of sulfurous compounds in livestock is normally related to the environmental impact, since these compounds are one of the main reasons responsible for the malodor in animal production facilities.^58^ Additionally, they can also be used as protein degradation markers.^59^

In this work, a simple methodology for the analysis of multiclass volatile metabolites in rumen fluid, urine, and feces of ruminants is presented. The method aimed for the metabolic characterization of these complex biological samples for the study of the rumen function. Several classes of compounds were characterized in the biological fluids of Holstein cows, the importance of which in ruminants’ digestion and metabolism was critically discussed. This work also introduces important steps on the state-of-the-art in the field, by introducing a simple and versatile analytical workflow to analyze noninvasive biological samples (urine and feces). This latter aspect is critical to facilitate the future monitoring of rumen function and animal metabolism in a noninvasive fashion, expanding the scope of the ruminant’s nutritional studies.

## Supporting Information Available

Figure S1: Chromatograms of a feces sample extracted with different acids (trichloroacetic acid, phosphoric acid, and hydrochloric acid); Table S1: Intraday precision (as relative standard deviation, %) and recovery values obtained for the spiking of the studied samples at 3 concentration levels (PDF)
