# Supplementary material for: A Novel Approach for Monitoring the Volatile Metabolome in Biological Samples from Ruminants through Miniaturized Liquid–Liquid Extraction and Multiclass Gas Chromatography Analysis
Source: J Agric Food Chem. 2022 Mar 17;70(12):3886–97. doi: 10.1021/acs.jafc.1c06662 (PMC9776526; doi:10.1021/acs.jafc.1c06662)
Supplement: Supplementary file 1 — jf1c06662_si_001.pdf [file jf1c06662_si_001.pdf]

A Novel Approach for Monitoring the Volatile Metabolome in Biological Samples from Ruminants through Miniaturized Liquid-Liquid Extraction and Multiclass Gas Chromatography Analysis

Liliana Cordeiro[a], Ana R. J. Cabrita[a], Hugo M. Oliveira[b], Margarida R. G. Maia[a], José A. Rodrigues[c], António J. M. Fonseca[a], Inês M. Valente[a,c]

[a]REQUIMTE, LAQV, ICBAS, Instituto de Ciências Biomédicas Abel Salazar, Universidade do Porto, Rua Jorge Viterbo Ferreira, 228, 4050-313 Porto, Portugal

[b]INL, International Iberian Nanotechnology Laboratory, Avenida Mestre José Veiga s/n, 4715-330 Braga, Portugal

[c]REQUIMTE, LAQV, Departamento de Química e Bioquímica, Faculdade de Ciências, Universidade do Porto, Rua do Campo Alegre 687, 4169-007 Porto, Portugal

Figure S1. Chromatograms of a feces sample extracted with different acids (trichloroacetic acid, phosphoric acid and hydrochloric acid).

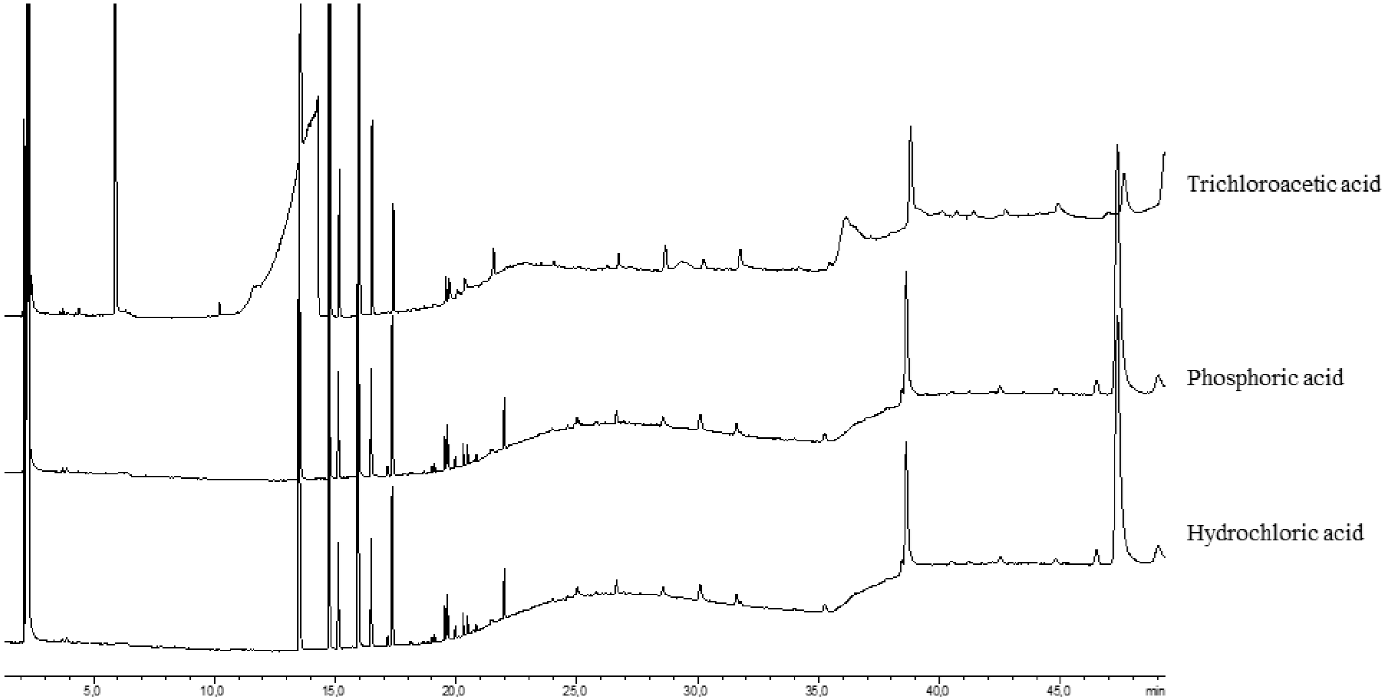

Table S1. Intraday precision (as relative standard deviation, %) and recovery values obtained for the spiking of the studied samples at 3 concentration levels.

Compounds	Added concentration (μmol L^{-1})	Rumen fluid		Urine		Feces	
		Intraday precision (%)	Recovery (%)	Intraday precision (%)	Recovery (%)	Intraday precision (%)	Recovery (%)
SHORT-CHAIN FATTY ACIDS							
Acetic acid	4.2×10^3	6	101 ± 29	6	109 ± 5	13	91 ± 5
	8.3×10^3	9	108 ± 13	2	84 ± 1	7	93 ± 23
	16.1×10^3	9	98 ± 1	1	107 ± 1	7	90 ± 20
Propionic acid	4.0×10^3	5	103 ± 11	12	89 ± 11	11	73 ± 27
	7.9×10^3	9	110 ± 14	2	109 ± 2	10	90 ± 3
	15.5×10^3	8	92 ± 19	2	98 ± 2	7	85 ± 4
isobutyric acid	0.3×10^3	2	95 ± 7	12	82 ± 14	8	73 ± 19
	0.7×10^3	8	89 ± 8	2	111 ± 2	9	84 ± 7
	1.3×10^3	7	85 ± 11	3	103 ± 3	8	100 ± 11
Butyric acid	3.9×10^3	3	106 ± 4	12	87 ± 10	10	103 ± 15
	7.7×10^3	8	98 ± 13	2	112 ± 2	10	84 ± 6
	15.0×10^3	7	88 ± 12	2	104 ± 3	6	104 ± 8
isovaleric acid	3.2×10^3	3	113 ± 3	12	86 ± 10	15	95 ± 8
	6.4×10^3	10	94 ± 8	2	113 ± 2	12	89 ± 11
	12.4×10^3	6	88 ± 7	3	105 ± 3	2	106 ± 1
Valeric acid	1.7×10^3	2	94 ± 3	11	87 ± 10	13	96 ± 10
	3.3×10^3	9	75 ± 9	2	114 ± 2	10	94 ± 8
	6.4×10^3	6	76 ± 6	3	106 ± 3	5	107 ± 5
Caproic acid	0.2×10^3	1	106 ± 2	5	104 ± 5	13	82 ± 11
	0.5×10^3	7	91 ± 11	2	106 ± 2	11	93 ± 5

	0.9 x 10^3	6	94 ± 10	3	106 ± 3	12	104 ± 6
ALDEHYDES							
Propanal	6.6	18	78 ± 1	3	73 ± 4	9	97 ± 8
	13.0	14	88 ± 4	16	88 ± 2	19	80 ± 15
	25.4	6	78 ± 4	8	71 ± 7	6	95 ± 5
Butanal	6.8	4	84 ± 1	8	100 ± 8	1	74 ± 1
	13.4	15	91 ± 2	9	92 ± 8	9	70 ± 5
	26.1	11	84 ± 10	10	95 ± 9	16	93 ± 9
Nona-2,4-dienal	6.5	10	105 ± 11	8	105 ± 8	15	82 ± 0
	12.9	13	92 ± 11	3	101 ± 3	11	86 ± 1
	25.1	5	85 ± 4	5	99 ± 5	3	94 ± 3
ALCOHOLS							
isopropanol (2-propanol)	6.4	3	108 ± 1	17	115 ± 15	8	108 ± 24
	12.7	9	96 ± 4	13	104 ± 15	26	101 ± 54
	24.7	5	108 ± 6	8	81 ± 5	20	95 ± 4
1-phenylethanol	6.0	4	111 ± 8	12	78 ± 9	12	87 ± 4
	11.8	5	94 ± 6	6	82 ± 3	7	76 ± 8
	23.1	11	92 ± 7	6	85 ± 3	2	99 ± 3
KETONES							
2-butanone	6.8	6	88 ± 11	2	69 ± 6	18	90 ± 2
	13.5	11	84 ± 4	14	68 ± 32	4	98 ± 17
	26.4	6	93 ± 8	5	68 ± 9	4	87 ± 2
ESTERS							
Methyl 2-methylbutyrate	6.4	5	76 ± 2	8	115 ± 9	9	79 ± 8
	12.6	14	85 ± 1	18	100 ± 1	9	94 ± 9
	24.7	8	86 ± 7	6	94 ± 5	4	98 ± 4
Ethyl phenylacetate	6.4	6	90 ± 2	3	98 ± 3	21	95 ± 2

	12.7	11	93	±	1	4	102	±	0	7	91	±	2
	24.8	6	89	±	6	4	97	±	4	4	104	±	5
PHENOLS													
4-methylphenol (*p*-cresol)	7.0	4	111	±	2	2	111	±	5	13	83	±	4
	13.8	7	102	±	12	5	98	±	8	4	93	±	6
	27.0	6	95	±	8	3	88	±	9	6	102	±	8
4-ethylphenol	6.4	6	101	±	3	3	73	±	11	11	80	±	10
	12.7	11	101	±	8	4	79	±	13	9	91	±	3
	24.8	7	99	±	8	2	83	±	3	3	106	±	4
3-ethylphenol	6.7	3	102	±	2	7	83	±	0	17	71	±	5
	13.2	13	102	±	4	2	94	±	3	12	85	±	13
	25.9	7	90	±	8	5	92	±	7	5	97	±	6
SULPHIDES													
Dimethyl sulphide	6.6	16	90	±	6	4	81	±	1	13	76	±	8
	13.1	20	102	±	3	5	105	±	5	19	96	±	10
	25.6	11	90	±	10	2	97	±	1	16	104	±	1
tert-butyl methyl sulphide	6.6	9	95	±	8	10	101	±	10	18	80	±	14
	13.1	11	92	±	6	2	95	±	2	9	93	±	4
	25.5	8	88	±	8	2	98	±	2	2	103	±	0
